# Nanoscale Waveguide Beam Splitter in Quantum Technologies

**DOI:** 10.3390/nano12224030

**Published:** 2022-11-16

**Authors:** Dmitry Makarov, Ksenia Makarova, Yuliana Tsykareva, Sergey Kapustin, Anastasia Kharlamova, Eugeny Gusarevich, Andrey Goshev

**Affiliations:** Department of Fundamental and Applied Physics, Northern (Arctic) Federal University, nab. Severnoi Dviny 17, 163002 Arkhangelsk, Russia

**Keywords:** beam splitter, nanosize, photons, wave function, non-monochromatic photons, reflection coefficient, transmission coefficient

## Abstract

Usually in quantum optics, the theory of large- and small-scale waveguide beam splitters is the same. In this paper, it is shown that the theory of the nanoscale waveguide beamsplitter has a significant difference from a similar device, but of a larger scale. It is shown that the previously known theory of the waveguide beam splitter is a particular case of the theory presented here. The wave function at the output ports of the nanoscale beam splitter is analyzed. The results obtained are sensitive to the size of the beam splitter, the coupling parameter of the two waveguides, and the degree of nonmonochromaticity of the photons entering the first and second ports of the beam splitter. The results are important for quantum technologies using a nanosized beam splitter.

## 1. Introduction

**Figure 1:**
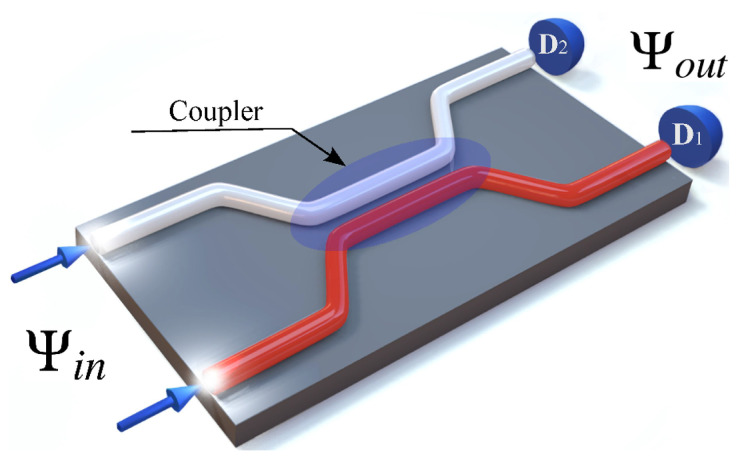
3D representation of the waveguide BS. Photons (in the general case nonmonochromatic) fall on the input ports BS. At the output ports of the BS are detectors $D_{1},D_{2}$ registering photons. The figure highlights the coupling region of the waveguide, where the electromagnetic fields from ports 1 and 2 overlap.

It is well known that the waveguide beam splitter (BS) is one of the main devices in quantum technologies [1,2,3,4,5,6,7]. Waveguide BS can be applied in many areas of modern quantum technologies, such as quantum metrology [8], quantum information [9], and linear optical quantum computing (LOQC) [4,5,6,7]. Additionally, using BS, you can create quantum entanglement between the input modes of electromagnetic fields [3,10,11], simulate quantum transport [12], and determine the degree of identity of photons [10,13], etc. This device has a great prospect of application due to its small size. This, in turn, leads to the fact that at small sizes new phenomena can arise that are not inherent in similar devices, but on a large scale. It is usually considered that the main characteristics of a waveguide BS are the reflection coefficients R and transmission coefficients T, which are constant values. This means that by setting these parameters one can always obtain the required characteristics at the output ports of the BS [3,8,11,14,15,16,17]. Previously, the results obtained did not qualitatively depend on the size of the BS, although the fact of a qualitative change in the properties of photons at the output ports of the BS depending on the dimensions of the BS (more precisely, the coupling region in the BS, see Figure 1) is quite obvious. Indeed, if the size of the BS becomes comparable in the order of magnitude with the wavelength of photons incident on ports 1 and 2 of the BS, then this should affect the properties of the photons at the output ports of the BS.

It was shown in [18,19] that using a BS based on coupled waveguides, i.e., the waveguide BS Hong–Ou–Mandel (HOM) effect, may not be performed even if *R* = *T* = 1/2 and the photons used are identical. It was also pointed out in these papers that in the main, such changes in the example of the HOM effect appear for a sufficiently small waveguide BS. Further development of the theory of coupled waveguides showed that the previously known theory is not always applicable, not only for the HOM effect but also for a waveguide BS in general [20,21]. This is due to the fact that in old theories the coefficients *R* and *T* are always constant for waveguide BSs, and as shown in [20,21,22,23], this is not the case for the non-monochromatic photons incident on 1 and 2 BS ports. These coefficients are dependent on the frequencies of the incident photons on the ports of the BS.

In this paper, we show that the properties of nonmonochromatic photons at the output ports of the BS strongly depend on its size in the case of the nanowave BS. In this case, as the size of the BS increases, the properties of the photons at the output ports become constant and do not depend on its size. In the case of monochromatic photons, the properties of the BS are the same regardless of its size.

Furthermore, we will use the atomic system of units: *ℏ* = 1; |e| = 1; $m_{e}$ = 1, where *ℏ* is the Dirac constant, *e* is the electron charge, and $m_{e}$ is the electron mass.

## 2. Materials and Methods

Consider a waveguide BS of arbitrary size. At the input ports of such a BS two modes of the electromagnetic field fall that are described by the wave function $\Psi_{in}$. At the output ports of this BS (see Figure 1), photons described by the wave function $\Psi_{out}$ are registered.

It has been shown in [20,21,22] that photon states at output ports of a waveguide BS can be represented by

(1) $$\begin{array}{c} \Psi_{out}=\sum_{k=0}^{s_{1}+s_{2}}\int \phi \left(\omega_{1},\omega_{2}\right)c_{k,p}|k,s_{1}+s_{2}-k\rangle d\omega_{1}d\omega_{2},\\ c_{k,p}=\sum_{n=0}^{s_{1}+s_{2}}A_{n,s_{1}+s_{2}-n}^{s_{1},s_{2}}A_{n,s_{1}+s_{2}-n}^{*k,p}e^{-2in\;\arccos\left(\sqrt{1-R}\sin\phi \right)},\;\\ A_{n,m}^{k,p}=\frac{\mu^{k+n}\sqrt{m!n!}}{{\left(1+\mu^{2}\right)}^{\frac{n+m}{2}}\sqrt{k!p!}}P_{n}^{\left(-(1+m+n),m-k\right)}\left(-\frac{2+\mu^{2}}{\mu^{2}}\right),\\ \mu =\sqrt{1+\frac{1-R}{R}\cos^{2}\phi }-\cos\phi \sqrt{\frac{1-R}{R}},\\ R=\frac{\sin^{2}\left(\Omega t_{BS}/2\sqrt{1+\varepsilon^{2}}\right)}{\left(1+\varepsilon^{2}\right)};\;T=1-R;\;\cos\phi =-\varepsilon \sqrt{\frac{R}{T}};\;\varepsilon =\frac{\omega_{2}-\omega_{1}}{\Omega }, \end{array}$$

where $|k,s_{1}+s_{2}-k\rangle =|k\rangle |p\rangle$ is the state of the photons at the output ports of the BS; $s_{1}$ and $s_{2}$ are the input number of photons in modes 1 and 2, respectively; $\phi (\omega_{1},\omega_{2})$ is the joint spectral amplitude (JSA) of the two-modes wavefunction ($\int |\phi \left(\omega_{1},\omega_{2}\right){|}^{2}d\omega_{1}d\omega_{2}=1$); Ω is a certain frequency characterizing the BS; and $t_{BS}=L/v$ is the time of interaction of photons in the BS (*L* is the length of the binding region in the waveguide BS, and *v* is the speed of light propagation in the waveguide). In Equation (1), the coefficients *R* and *T* are reflection and transmission coefficients, respectively. It should be added that Equation (1) is not only responsible for the case of non-monochromatic photons falling on the BS input ports but also monochromatic ones. For this purpose, it is sufficient to set JSA (the parameter responsible for the spectral width) $\phi (\omega_{1},\omega_{2})$ to zero. It should be added that in the case of monochromatic and identical photons, the obtained expressions coincide with [1], where $R=\sin^{2}\left(Cz\right)$, ϕ=π/2, C=Ω/(2v ) is the coupling constant between neighbouring waveguides. In addition, it is worth adding that in reality there are no monochromatic photons and the analysis must be based on total Equation (1). In addition, the more coupling in the waveguide, the higher the value of Ω and vice versa. Thus, we can adjust the coupling in the waveguide by changing Ω.

To study the output states of photons at the ports of the BS, we will study two characteristics: the probability $P_{k,p}$ of detecting *k* and *p* photons at 1 and 2 output ports of the BS, respectively. We will take into account, as shown in [20,21], that the number of photons is conserved, i.e., $s_{1}+s_{2}=k+p$. An extremely important characteristic in quantum technology and information is the quantum entanglement of photons. To characterize quantum entanglement, the Von Neumann entropy $S_{N}$ will be used, which as shown in [20,21]; it will be determined by $S_{N}=-\sum_{k}P_{k,s_{1}+s_{2}-k}\ln\left(P_{k,s_{1}+s_{2}-k}\right)$. To find the probability $P_{k,p}$ we can use the previously known [20,21] expression

(2) $$\begin{array}{c} P_{k,s_{1}+s_{2}-k}=\int {\left|\phi \left(\omega_{1},\omega_{2}\right)\right|}^{2}\lambda_{k}\left(R\right)d\omega_{1}d\omega_{2},\;\;\lambda_{k}\left(R\right)={\left|c_{k,s_{1}+s_{2}-k}\right|}^{2}. \end{array}$$

## 3. Results

The joint spectral amplitude (JSA) $\phi (\omega_{1},\omega_{2})$ must be determined in order to present and analyze the results of the calculations. We will use the best known form

(3) $$\begin{array}{c} \phi_{i}\left(\omega_{i}\right)=\frac{1}{{\left(2\pi \right)}^{1/4}\sqrt{\sigma_{i}}}e^{-\frac{{\left(\omega_{i}-\omega_{0i}\right)}^{2}}{4\sigma_{i}^{2}}}, \end{array}$$

where $\omega_{0i}$ is the mean frequency and $\sigma_{i}^{2}$ is the dispersion. Next, we will use the $\omega_{0i}/\sigma_{i}\gg 1$ condition, which is applicable to most photon sources. It should be added that the form Equation (3) is the best known function and corresponds to the distribution of photons in Fock states.

Further, we will assume that the incident photons at ports 1 and 2 of the BS are identical, i.e., $\sigma_{1}=\sigma_{2}=\sigma$ and $\omega_{02}=\omega_{01}=\omega_{0}$. The use of identical photons in quantum technologies is one of the important properties since with such an identity quantum the coherence and quantum entanglement of photons begin to appear. This is easy to show qualitatively using Equation (1) using the expression for the reflection coefficient *R*. Indeed, if $\omega_{2}-\omega_{1}\gg \Omega$ is chosen, then the coefficient R≪1, which leads to the propagation of photons along their original waveguides, and the coupled waveguide does not exhibit the properties of a BS. In this case, it is not difficult to show that the main characteristics of the electromagnetic field at the output ports of the BS will depend only on two parameters σ/Ω and $L/L_{BS}$, where $L_{BS}=v/\Omega$. The value of $L_{BS}$ plays an important role in the BS; firstly, if $L\ll L_{BS}$, then the properties of the BS are not observed, i.e., photons in waveguides propagate unchanged; secondly, if $L≳L_{BS}$, then the main characteristics of photons at the output ports of the BS have a non-trivial dependence. In the case of $L≳L_{BS}$, a qualitative analysis can be carried out if we consider the reflection coefficient *R* in (1) and consider the photons to be identical. In this case, during the analysis, the parameter $L_{const}=L_{BS}\Omega /\sigma =v/\sigma$ will appear at which if $L\gg L_{const}$ the main characteristics of photons at the output ports of the BS do not depend on of length *L*. For other values of *L*, the dependence of the main characteristics of photons at the output ports of the BS is rather complicated. It should be added that the length $L_{BS}$ has a simple physical meaning; it is the characteristic coupling length of the waveguide, i.e., with less than this length, the connection between waveguides is not observed.

**Figure 2:**
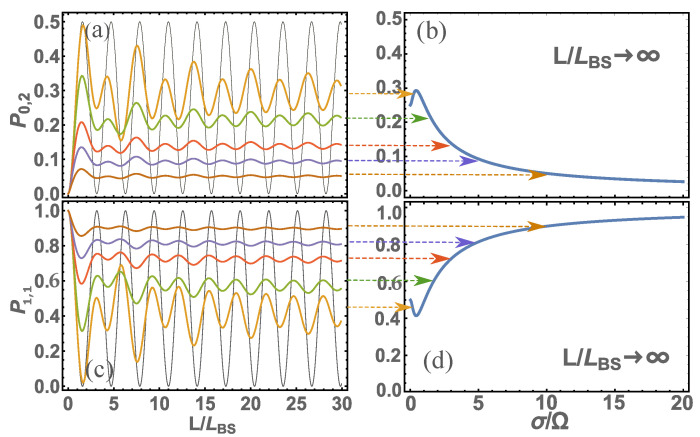
The calculation is presented: (**a**) probabilities $P_{0,2}$ of detecting 2 photons at the second detector and 0 of photons at 1 detector (with $P_{0,2}=P_{2,0}$ ) at different parameters σ/Ω={0,1/2,3/2,3,5,10} (respectively, the color of the graphs in the figure: {black, yellow, green, red, blue, brown}) depending on the dimensionless BS length $L/L_{BS}$; (**b**) the same but with larger dimensions of the beamsplitter, i.e., at $L/L_{BS}\to \infty$; (**c**) the same as in (**a**) but only for the probability $P_{1,1}$ of one photon detected at each detector; (**d**) the same as in (**b**) but only for the probability $P_{1,1}$.

**Figure 3:**
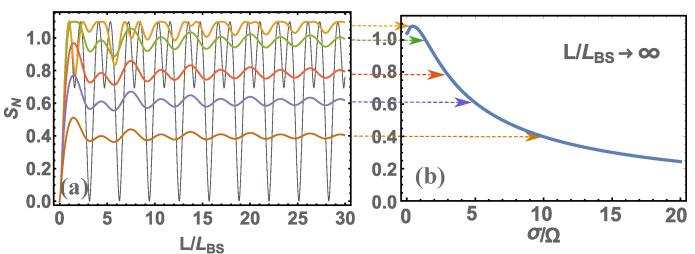
The calculation is presented: (**a**) quantum entanglement $S_{N}$ at different parameters σ/Ω={0,1/2,3/2,3,5,10} (respectively, the color of the graphs in the figure: {black, yellow, green, red, blue, brown}) depending on the dimensionless BS length $L/L_{BS}$; (**b**) the same but with larger dimensions of the BS, i.e., at $L/L_{BS}\to \infty$.

Next, we present an illustration of the calculation of the probability of detecting photons at 1 output port of the BS in the case when one photon each falls on the input ports, i.e., $s_{1}=1,s_{2}=1$ (or |1,1〉), see Figure 2. Let us also present, for the same data ($s_{1}=1,s_{2}=1$), the calculation of the quantum entanglement of photons at the output of the BS Figure 3. The calculations were performed for various cases of nonmonochromaticity of incident photons.

**Figure 4:**
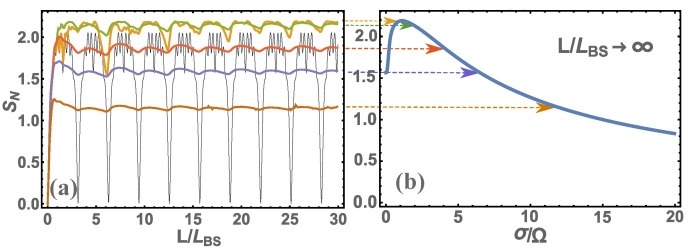
The calculation for the initial state |4,4〉 is presented: (**a**) quantum entanglement $S_{N}$ at different parameters σ/Ω={0,1/2,3/2,3,5,10} (respectively, the color of the graphs in the figure: {black, yellow, green, red, blue, brown}) depending on the dimensionless BS length $L/L_{BS}$; (**b**) same but at larger BS size, i.e., at $L/L_{BS}\to \infty$.

From Figure 2 and Figure 3, we can see that the patterns are qualitatively the same. This means that the general analysis of these regularities will be similar regardless of whether we consider probabilities or quantum entanglement. From the presented figures, it is clearly visible that at small values of $L/L_{BS}$ the main characteristics tend to zero, and at $L/L_{BS}\to \infty$ tend to a constant value, i.e., the main characteristics cease to depend on the light splitter length. As shown above, there is a more stringent condition for determining the transition of the basic characteristics to a constant value is $L\gg L_{BS}\Omega /\sigma =v/\sigma$. If you look at these figures, you can see that this condition is indeed satisfied. You can also see that if the photons are monochromatic, i.e., v/σ→∞, then L→∞. In other words, in the case of monochromatic photons, the main characteristics of photons at the output ports of the BS will never be constant and will depend on the length of the BS. Of course, in reality there is no such thing since there are no completely monochromatic photons, which means that at a certain length of the BS the main characteristics will always be constant. Thus, we come to the definition of the main parameter $L_{const}=v/\sigma$, which determines the characteristic length of the BS; if this is exceeded, the main characteristics of photons at the output ports of the BS will become constant. Although here we have presented calculation results for |1,1〉 states, the same will be qualitatively true for other initial states of photons at BS input ports. For example, let us give the results of quantum entanglement calculations for $s_{1}=4,s_{2}=4$ (or |4,4〉), see Figure 4. Here, we do not present the probability of detecting photons in different states at the BS output ports since there are many such probabilities and it only makes sense to analyze quantum entanglement. We should add that our consideration of the initial state |4,4〉 corresponds in part to one of the Holland–Burnett (HB) $s_{1}=s_{2}=s$ (or |s,s〉) states [24], which is of great interest in various fields of physics, for example, in quantum metrology [8,25].

Typically, quantum technologies use optical photons with dispersion $\sigma ∼\{10^{13}-10^{14}\}$ rad/s. If we estimate the length $L_{const}$, we get $∼\{10^{3}-10^{4}\}$nm. This is the size at which units fit the order of the optical wavelengths. If higher-frequency radiation (e.g., ultraviolet) is used, these dimensions will be reduced and the sizes will be comparable to tens and hundreds of nanometers. If we use lower-frequency radiation (e.g., infrared), the sizes will be tens and hundreds of micrometers. Thus, when using nanoscale waveguides, the main characteristics of photons at the output ports of the BS will be sensitive to its size and will always depend on it. Moreover, these characteristics will be quite hard to predict (see Figure 2, Figure 3 and Figure 4) and have not only an oscillatory nature but also will change the amplitude of these characteristics. To determine these characteristics, it is necessary to carry out a calculation using Equation (1).

For a complete analysis of such BS, we need to determine the length $L_{BS}=v/\Omega$. As shown in [18,19], the frequency can be within $\Omega =\{10^{14}-10^{17}\}$ rad/s depending on what the waveguide consists of and how the waveguides are connected together. In any case, $L_{BS}<L_{const}$, which means that there is always a region of transition from zero values of the main characteristics to values when they do not depend on the length of *L* (for example, see Figure 2, Figure 3 and Figure 4).

## 4. Conclusions

Thus, it is shown that the nanosized beam splitter exhibits properties that are not manifested in large-sized BS. The boundary when the waveguide should be considered large is determined when the coupling length of the waveguide $L\gg L_{const}=v/\sigma$. Additionally, the boundary when the coupled waveguide does not exhibit the properties of a BS is determined when $L\ll L_{BS}=v/\Omega$. The properties of the BS here mean the main characteristics of photons at its output ports are the probabilities of detecting photons at ports 1 and 2, as well as quantum entanglement. It is shown that the properties of the BS do not depend on which characteristic we consider; the probability; or the quantum entanglement. All of these characteristics can be calculated using Equation (1). For example, we performed calculations for the initial states of photons falling on the input ports of the BS at |1,1〉 and |4,4〉. Regardless of these calculations, all conclusions are also suitable for arbitrary states $|s_{1},s_{2}\rangle$ since a qualitative analysis can be performed in a general form that leads to the same conclusions as those for the calculations at |1,1〉 and |4,4〉. The presented study is important since it was previously believed that the properties of photons at the output ports of waveguide BS do not depend on the degree of nonmonochromaticity of photons and that the dependence is the same for any size of BS. In this paper, it is shown that the properties of the photons at the output ports of a BS change significantly depending on the degree of nonmonochromaticity of the photons and at small sizes of BS (nanoscale waveguide BS) compared to previous results. Moreover, our theory is general since all of the results of the previous theory are a special case of the theory presented in this article. The potential application of the results obtained in this work is similar to the use of BS in quantum technologies (see introduction). This result should be used when reducing the waveguide BS to the nanoscale. The results can also be used to increase the quantum entanglement of photons at the output ports of the BS since, as shown above, at σ/Ω≈1 the quantum entanglement is close to its maximum value [21,22]. Indeed, it is well known that the maximum quantum entanglement for the von Neumann entropy is $S_{N}=\ln\left(1+N\right)$ when *N* is the total number of photons in a two-part system, e.g., [15,26], where $N=s_{1}+s_{2}$ [15].

It should be added that more and more attention is now being paid to waveguide BSs and their counterparts, with new properties being identified. For example, these include waveguide lattices and photonic lattices [27,28], photonic waveguide BS based on negative-index media [29], planar hyperbolic waveguide [30], and multiple linear-crossing metamaterials for directional refraction [31]. The nanoscale waveguide BS presented in this paper is a continuation of research in this area for quantum technology development.

## Data Availability

Send a request to the corresponding author of this article.
